# Wind turbine proximity and health-related quality of life in Germany 2002 to 2022

**DOI:** 10.1038/s41598-026-56835-5

**Published:** 2026-06-14

**Authors:** Gundi Knies, Jens Jetzkowitz

**Affiliations:** 1 Thünen-Institute of Rural Studies, Bundesallee 64, 38116 Braunschweig, Germany; 2 Institute of Sociology, Martin Luther University Halle-Wittenberg, 06099 Halle (Saale), Germany; 3 Section Methods of Empirical Social Research and Statistics, Faculty of Social Sciences and Humanities, Helmut Schmidt University, Post box 70 08 22, 22008 Hamburg, Germany

**Keywords:** Wind energy, Health-related quality of life, Wellbeing, Spatial analysis, Rural areas, Difference-in-differences, Engineering, Environmental sciences, Environmental social sciences, Environmental studies

## Abstract

The expansion of wind energy has intensified debate about potential impacts on the health and wellbeing of nearby residents, yet empirical evidence remains mixed. Using data from the German Socio-Economic Panel (SOEP) linked to administrative records on onshore wind turbines, we analyse changes in health-related quality of life (HRQoL) associated with turbine construction in Germany between 2002 and 2022. We implement a quasi-experimental matched difference-in-differences design among residential non-movers, focusing on turbines with hub height ≥ 50 m and proximity bands from 1.5 km to 6 km. We find no evidence of adverse average HRQoL changes associated with turbine presence within 6 km in this setting for matched residential non-movers over the observed pre-post interval. Secondary analyses indicate heterogeneity by turbine characteristics and cumulative exposure, including patterns consistent with lower mental HRQoL where turbine density is higher. These results are interpreted cautiously, given multiple related specifications and limitations in assessing pre-trends with biennial HRQoL measurement. Overall, the findings highlight the importance of cumulative exposure and turbine characteristics for assessing the local health implications of wind energy deployment.

**Supplementary Information:**

The online version contains supplementary material available at 10.1038/s41598-026-56835-5.

## Introduction

The transition from carbon-based, non-renewable energy sources to renewable alternatives is well underway across Europe, with wind energy playing a central role through the expansion of large-scale technical infrastructure^1,2^. Wind power is widely recognised as an effective means of mitigating climate change^3–5^, and policy instruments have proven successful in accelerating its deployment^6–9^. Consequently, the science-informed public debate has largely shifted from questioning whether wind energy should be expanded to addressing where wind turbines should be sited and at what distance from residential areas^10^. This shift reflects growing local resistance to wind turbine installations, which today is less focused on the technology per se and increasingly centred on potential health impacts and perceived reductions in quality of life^11–13^.

While concerns about health and well-being have become central to debates at the intersection of environmental policy, public health, and the social acceptance of wind energy infrastructure, the existing evidence suggests a multifaceted, still-ambiguous configuration of cause-and-effect relationships. This complexity calls for a causal inference perspective that treats wind turbine construction not as a singular determinant of health outcomes, but as an insufficient yet potentially contributory condition within a broader constellation of factors affecting physical and mental health^14^. Within the existing literature, reported impairments in physical and mental health in the vicinity of wind turbines are commonly linked to a constellation of background conditions, including direct environmental nuisances near residential areas, potential physiological effects of infrasound below the threshold of conscious perception, and expectation-driven responses such as nocebo effects.

A substantial body of research has examined associations between residential proximity to wind turbines and health-related outcomes, with annoyance emerging as the most consistent finding. Multiple studies report links between turbine exposure and annoyance, sleep disturbance, and psychological distress (e.g.,^15^), and some document lower overall, physical, and environmental quality of life among residents living near wind turbines (e.g.,^16^). At the same time, evidence for broader or clinically relevant health effects remains mixed and highly dependent on study design and outcome measures^17,18^. Systematic reviews generally conclude that, when turbines are appropriately sited, noise exposure, shadow flicker, electromagnetic fields, and low-frequency noise are unlikely to cause direct physiological harm, although annoyance persists as a recurrent and robust outcome^19,20^. More recent syntheses corroborate this pattern, finding consistent evidence for noise-related annoyance and risk perception but limited and inconclusive evidence for effects on sleep, mental health, or overall health-related quality of life^10,21,22^. Importantly, much of this literature relies on cross-sectional data, selected samples, or short-term exposures, constraining causal inference and limiting insights into longer-term wellbeing trajectories.

In parallel, infrasound has been proposed as a distinct pathway through which wind turbines may affect human health, yet empirical findings remain highly contested. While some studies suggest associations between turbine-generated infrasound or low-frequency noise and health-related symptoms^23,24^, others find no evidence of health impairment attributable to turbine noise or infrasound exposure^25–27^. Disagreement extends beyond health outcomes to questions of audibility, perceptibility, and relevant distance thresholds. In Germany, regulatory frameworks typically require setback distances between 600 m and 1 km and subject turbines above 50 m to permitting procedures intended to prevent harmful environmental effects^28,29^. Experimental evidence suggests that low-frequency noise at very short distances may influence physiological markers such as heart rate variability^30^, whereas field measurements suggest that turbine-related infrasound becomes difficult to distinguish from background noise beyond approximately 700 m^31^. Other studies report audibility at distances of several kilometres^32^. Taken together, these inconsistencies underscore the difficulty of isolating single biophysical mechanisms and point to the need for research designs that assess health outcomes under real-world exposure conditions without presupposing a specific causal pathway.

Beyond biophysical exposure pathways, a growing literature highlights the role of expectations, attitudes, and social context in shaping health responses to wind turbines. Empirical studies demonstrate that negative expectations and beliefs about wind turbines can contribute to annoyance and other health complaints via nocebo effects^33,34^. Such processes should not be dismissed as merely psychological artefacts: consistent with the Thomas theorem, subjective definitions of situations can have tangible consequences for health and wellbeing. Research further shows that visual impact, perceived fairness of planning and siting processes, and feelings of loss of control are important predictors of annoyance and stress responses, often exceeding the explanatory power of objective noise levels alone^35,36^. These findings suggest that health-related outcomes may arise from complex interactions between physical exposure, social meaning, and individual perception.

Taken together, the literature points to a fragmented and methodologically heterogeneous evidence base in which annoyance, contested biophysical exposures, and expectation-driven responses are closely intertwined. This complexity complicates causal attribution and limits the ability of existing studies to inform policy-relevant questions, such as how close wind turbines can be located to residential areas without adversely affecting health-related quality of life (HRQoL). To address these limitations, we adopt a quasi-experimental approach^37^ that exploits temporal and spatial variation in wind turbine construction in Germany. By linking high-resolution installation data with independently collected longitudinal health measures from the German Socio-Economic Panel (SOEP), we follow the same individuals over time and examine whether changes in local turbine exposure are associated with within-person changes in physical and mental health under real-world conditions. Because HRQoL is observed in a general population panel rather than in a turbine-specific survey, the design is less vulnerable to selection or response patterns driven by awareness of local wind-energy conflicts. Descriptively, regressions based on the full SOEP sample show a pronounced negative association between proximity and physical health (the SF-12 Physical Component Summary score, PCS), whereas estimates for mental health (the SF-12 Mental Component Summary score, MCS) are smaller and not statistically distinguishable from zero (Supplementary Table S1). These full-sample associations are difficult to interpret causally because they do not isolate treated and control observations around turbine commissioning and may reflect time-varying local factors correlated with turbine siting and residential selection.

This motivates our quasi-experimental strategy. Specifically, we focus on short- to medium-run within-person changes around turbine commissioning using a matched difference-in-differences design among residential non-movers, comparing changes in HRQoL between individuals who experience first-time turbine commissioning nearby and matched controls who remain unexposed within the near surrounding area. At the same time, we interpret the estimates cautiously, as causal interpretation still depends on assumptions about remaining time-varying confounding and potential spillovers. Our study is closely related to recent SOEP-based research on wind turbines and health and subjective wellbeing outcomes^18^. We complement this literature by implementing a matched two-period difference-in-differences design among residential non-movers with explicit treatment, control and ban radius restrictions, and by centring the main analysis on modern installations (hub height ≥ 50 m) across pre-specified distance bands, motivated by the German planning context. Taken together, this design strengthens inference relative to cross-sectional or turbine-specific survey designs while retaining a cautious scope of inference: short- to medium-run HRQoL changes among residential non-movers under real-world exposure conditions.

## Methods

### Health-related quality of life data

We use data from the German Socio-Economic Panel (SOEP, v39) covering the period 2002–2022^38^. The SOEP is a nationally representative longitudinal survey of private households in Germany, surveying approximately 30,000 individuals in 11,000 households annually. Since 2000, geocoded residential information has been available, enabling linkage to fine-grained spatially referenced data. Due to data protection regulations, analyses combining survey data and geographic coordinates were conducted in a secure environment at DIW Berlin.

We measure health-related quality of life (HRQoL) using the Short Form-12 Health Questionnaire (SF-12)^39^, which has been collected biennially in the SOEP since 2002. The SOEP provides norm-based Physical and Mental Component Summary scores (PCS and MCS) standardised to the German population in 2004 and scaled from 0 to 100, with higher values indicating better HRQoL. A score of 50 represents the mean of the reference population, and a 10-point difference corresponds to one standard deviation^40^. PCS summarises overall physical health and functioning, whereas MCS summarises overall mental health and wellbeing. As validated population-health summary measures, SF-12 PCS and MCS are widely used in epidemiological research and have also been applied in neighbourhood-effects studies examining associations between HRQoL and perceived neighbourhood problems and other contextual stressors.

### Wind turbines data

Wind turbine data were obtained from the Core Energy Market Registry (MaStR), which records all electricity-generating facilities in Germany^41^. We extracted geographic coordinates, commissioning and decommissioning dates, hub height, turbine type, and installed capacity for all onshore wind turbines. The analytical dataset covers turbines commissioned between January 2002 and December 2022.

To ensure replicability, we use the first archived version of the MaStR (dated 1 April 2023). In addition, we used a more detailed daily extract of the MaStR dated 13 March 2023, which contains site addresses, land parcel identifiers, and additional turbine-specific information. This information was used to validate and correct missing or implausible geographic coordinates (e.g., coordinates located outside Germany, multiple turbines assigned to identical locations, or missing coordinates).

Missing or inconsistent information on turbine type was corrected using publicly available turbine specifications, drawing on manufacturer documentation and wind park operator information. Turbine type corrections were based on installed capacity and rotor diameter, which are nearly always reported in the registry and are less error-prone than hub height, which is often not finalised prior to installation due to site-specific considerations.

Missing information on hub height was corrected where possible using publicly available site-level information (e.g., wind park operator websites) and turbine type specifications. For turbine models produced with a single standard hub height, this value was assigned directly. For turbine types manufactured with multiple possible hub heights, hub height was assigned by randomly selecting one of the manufacturer-specified options with equal probability. Importantly, for the vast majority of these cases, all admissible hub heights for a given turbine type lay either entirely above 100 m or entirely below 50 m, such that random assignment did not affect classification with respect to the hub height thresholds used in the analysis. For the remaining cases with missing hub height information, values were imputed using data from the Renewable Energy Monitor^42^, which assigns hub height as the mean observed value for the corresponding turbine type. These procedures were limited to resolving missing or implausible values and did not affect commissioning dates or installed capacity.

After data cleaning, the sample comprises 23,600 onshore wind turbines relevant to the study. All spatial exposure measures are based on the corrected turbine coordinates and characteristics. Due to the structure of the secure data linkage, turbine-level identifiers are not available in the analytical dataset, precluding identification of specific turbines as individual treatments.

### Treatment, treatment radius and ban radius specification

The potential health effects of wind turbines are not well established, and no generally accepted exposure threshold exists. In Germany, there is no uniform nationwide regulation governing minimum distances between wind turbines and residential areas. However, installations exceeding 50 m in height are subject to permitting procedures under the Federal Building Code (Baugesetzbuch), which aim to prevent harmful environmental effects on humans and nature. In practice, minimum setback distances from residential areas typically range between 600 m and 1 km, depending on federal state regulations^28,29^.

In environmental impact assessments, the spatial extent considered in the analysis of wind turbine effects depends on turbine characteristics and on the protected good under consideration. Guidance used in German licensing procedures, therefore, specifies investigation radii that may extend several kilometres beyond minimum setback distances for selected receptors. In sensitive cases, practical assessments may employ investigation radii of up to 6 km, e.g.,^43^. Such distances define the spatial scope within which potential impacts are systematically assessed; they do not imply that effects are expected to occur across the entire area.

Similarly, several epidemiological studies define exposure using radii of up to 6 km when linking residential locations to modelled exposure, e.g.,^18,44–46^. These radii serve a methodological purpose: they identify the population potentially exposed and allow aggregation of contributions from multiple surrounding turbines in exposure metrics. They should therefore be interpreted as investigation or sampling radii rather than as evidence of a corresponding effect radius.

In public and political discussions, however, investigation radii and effect radii are sometimes treated as interchangeable concepts, even though they are analytically distinct. At the same time, exposure pathways most plausibly relevant for residents - such as audible noise (including low-frequency components), annoyance and sleep disturbance, shadow flicker, aviation lighting and visual intrusion - are expected to be strongest at shorter distances and to attenuate with distance. In settings with multiple turbines, cumulative exposure may nevertheless become relevant over somewhat larger spatial scales.

Against this background, our primary specification focuses on turbines with hub height ≥ 50 m and defines exposure using circular treatment radii around respondents’ residences. In geocoded difference-in-differences settings, the choice of treatment and control distances, and the handling of spatial spillovers, are important design decisions^47^. We therefore pre-specify a set of treatment radii and implement a ban radius to reduce spillover contamination between treatment and control observations. Specifically, we define treatment using radii up to 6 km in 1 km increments. We use 1.5 km as the nearest feasible near-field treatment radius because the number of treated cases within 1 km is too small to support stable inference in the matched residential non-mover design. In earlier estimates, we examined treatment radii at 500 m intervals; the qualitative conclusions were unchanged. We report 1 km steps up to 6 km to preserve interpretability and because restrictions in the secure data environment limit the number of results that can be exported from the analysis setting.

To provide transparency on sensitivity to turbine definitions used in planning practice and to measurement issues in registry fields, we additionally consider three secondary turbine classifications: (i) all onshore wind turbines irrespective of size, (ii) turbines with a hub height of at least 100 m, and (iii) turbines with a capacity of at least 500 kW as an alternative proxy for installation size given known uncertainties in hub-height reporting in the MaStR. These secondary classifications are treated as complementary rather than as alternative primary specifications.

To ensure a clear separation between treatment and control observations, we define the control group using a control ring between 10 and 15 km, following previous SOEP-based research^17^. This control definition ensures that both groups reside in areas where wind turbine siting is plausible while remaining unexposed in the near surrounding area. Given this control-ring definition, we implement a 10 km exclusion (“ban”) radius to reduce contamination from nearby turbines outside the treatment radius^17^. Specifically, for a given treatment radius $\:r$ (1.5–6 km), we exclude observations if any turbine is present in the ban ring between $\:r$ and 10 km during the observation window used for the pre/post comparison. This restriction is a pragmatic compromise: a smaller exclusion boundary (e.g., 8 km) would admit more “near-exposed” observations into the comparison set and increase contamination of the control condition, likely attenuating estimates toward zero, whereas a larger boundary would further reduce contamination but materially reduce the pool of eligible matched non-movers and thus statistical power. Throughout, treated observations are defined by the commissioning of at least one qualifying turbine within $\:r$ between $\:{t}_{0}$ and $\:{t}_{1}$ (and operational at $\:{t}_{1}$), whereas control observations have at least one turbine in the 10–15 km control ring and none within 10 km during the relevant observation window.

### Allocation to quasi-experimental groups

We construct quasi-experimental treatment and control groups based on changes in wind turbine exposure between two SF-12 observation points, denoted $\:{t}_{0}$ (pre-treatment) and $\:{t}_{1}$ (post-treatment). Eligible individuals provided at least two valid SF-12 observations, did not change residence between $\:{t}_{0}$ and $\:{t}_{1}$, and had no wind turbines within a 10 km radius of their residence at baseline ($\:{t}_{0}$).

Individuals are assigned to the treatment group for radius $\:r$ if at least one wind turbine is commissioned within $\:r$ (1.5–6 km) between $\:{t}_{0}$ and $\:{t}_{1}$, is operational at $\:{t}_{1}$ and no other turbine is located in the corresponding ban ring spanning from $\:r$ to 10 km during the relevant observation window. Individuals are assigned to the control group if at least one turbine is located in the match radius between 10 and 15 km from their residence at $\:{t}_{1}$ and no turbine is present within 10 km at any point during the relevant observation window. This control definition ensures that both groups reside in areas where turbine construction is plausible and where broader locational characteristics are comparable, while maintaining an unexposed local environment within 10 km for the control group.

**Fig. 1:**
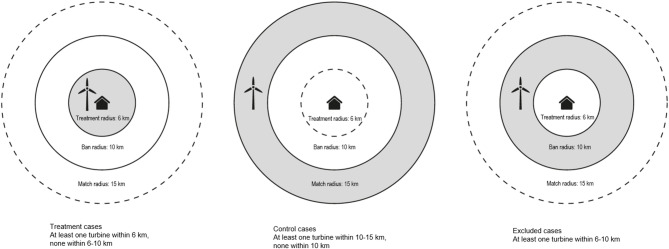
Illustration of allocation to treatment and control group based on placement of turbines in the treatment radius ($\:r$=6 km), ban radius (10 km) and match radius (15 km). Source: Own illustration.

For individuals with more than one eligible pair of SF-12 observations, we select the pair closest in time to the turbine construction event. Because SF-12 is collected every two years in the SOEP, the $\:{t}_{0}$ to $\:{t}_{1}$ interval is typically around two years, implying that the main difference-in-differences estimates capture short- to medium-run changes in HRQoL around commissioning rather than long-run adaptation. This two-period construction limits the scope for conventional event-study lead/lag diagnostics within the main analysis sample. Individuals who enter a treatment group later in the panel are excluded from serving as controls at earlier observation periods. Figure 1 illustrates the spatial definition of treatment and control areas and the resulting allocation to quasi-experimental groups.

### Empirical model

We estimate treatment effects using a matched two-period difference-in-differences (DiD) design among residential non-movers, implemented within an individual fixed-effects panel framework. The approach compares within-person changes in the outcome between treated and control individuals from the last pre-treatment SF-12 observation ($\:{t}_{0}$) to the first post-treatment SF-12 observation ($\:{t}_{1}$) around turbine commissioning.

The empirical model is specified as:

1 $$\:{y}_{it}={\alpha\:}_{i}+{\lambda\:}_{t}+{\beta\:}_{i}{WT}_{it}\:+{\epsilon\:}_{it}$$

where $\:y$ is the outcome for individual *i* at survey time *t*, $\:{\alpha\:}_{i}$ captures all time-invariant individual traits (individual fixed effects), $\:{\lambda\:}_{t}$ the period fixed effect, and $\:\epsilon\:$ the idiosyncratic error term. *WT*_*it*_ is a treatment indicator equal to one in the post-treatment period ($\:{t}_{1}$) for individuals whose residence experiences commissioning of at least one qualifying turbine within the treatment radius r between $\:{t}_{0}\:$and $\:{t}_{1}$ (and operational at $\:{t}_{1}$), and zero otherwise. By construction, treated observations satisfy the radius-specific ban-ring restrictions described above, and control observations have no turbines within 10 km at any point in the relevant observation window while having at least one turbine between 10 and 15 km. The coefficient β is the average DiD estimate for residential non-movers over the observed $\:{t}_{0}$ to $\:{t}_{1}$ interval.

To address observable baseline differences between treatment and control groups, we apply propensity score matching (nearest-neighbour 1:1 matching) at t_0_. Individual-level covariates include sex, age group (< 40 years, 40–49 years, 50–59 years, 60–69 years, ≥ 70 years), whether the respondent had any overnight hospital stays in the previous calendar year (yes/no), and the number of doctor visits in the three months preceding the interview at the last pre-treatment wave (none, 1–2 visits, more than two visits). Area-level covariates include the degree of rurality (non-rural, rural, very rural), whether the residence is located in a residential area (yes/no), and the proportion of municipal area used for road traffic, classified relative to rurality-specific averages. Covariate balance between matched treatment and control groups is satisfactory across all specifications (see Supplementary Table S2 and Supplementary Figure S1).

In further specifications, we examine exposure intensity within the treatment radius $\:r$. For this, we multiply the treatment dummy by (a) the number of operational turbines within $\:r$ at $\:{t}_{1}$ and (b) the inverse distance to the closest operational turbine within $\:r$ at $\:{t}_{1}$. These measures proxy cumulative exposure and proximity, but do not directly measure noise, flicker, or visibility. All models are estimated separately for the PCS and MCS scores defined above. Exposure is defined using distance buffers around residences rather than administrative boundaries, because turbines may be sited near municipal borders and affect residents in neighbouring jurisdictions. This also implies that no single administrative clustering level perfectly matches the spatial correlation structure; we therefore report individual-clustered standard errors and interpret marginal significance cautiously. Because we test closely related hypotheses across multiple distance bands, we report Benjamini-Hochberg false discovery rate (FDR) adjusted q-values in addition to conventional p-values^48^. Analyses are conducted using Stata version 18.

### Ethics approval and consent to participate

All methods were carried out in accordance with relevant guidelines and regulations. This study is based on secondary analyses of anonymised data from the German Socio-Economic Panel (SOEP). Participation in the original SOEP survey was voluntary and based on informed consent. Data collection and processing comply with applicable data protection regulations, including the EU General Data Protection Regulation (GDPR) and the German Federal Data Protection Act (Bundesdatenschutzgesetz). No separate institutional ethics approval is required for this secondary analysis.

## Results

**Fig. 2:**
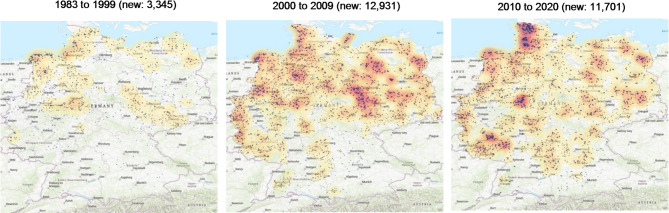
Expansion of wind turbines in Germany from 1983 to the end of 2020. Source: Core Energy Market Data Register (MaStR, v2023.04.01) with own corrections.

Figure 2 illustrates the expansion of wind turbines in Germany from 1983 to the end of 2020 using kernel density plots. The black spots on the map represent the exact locations of wind turbines present at any time during the depicted period; the heat map suggests where more wind turbines have been built than in the respective previous period.

**Fig. 3:**
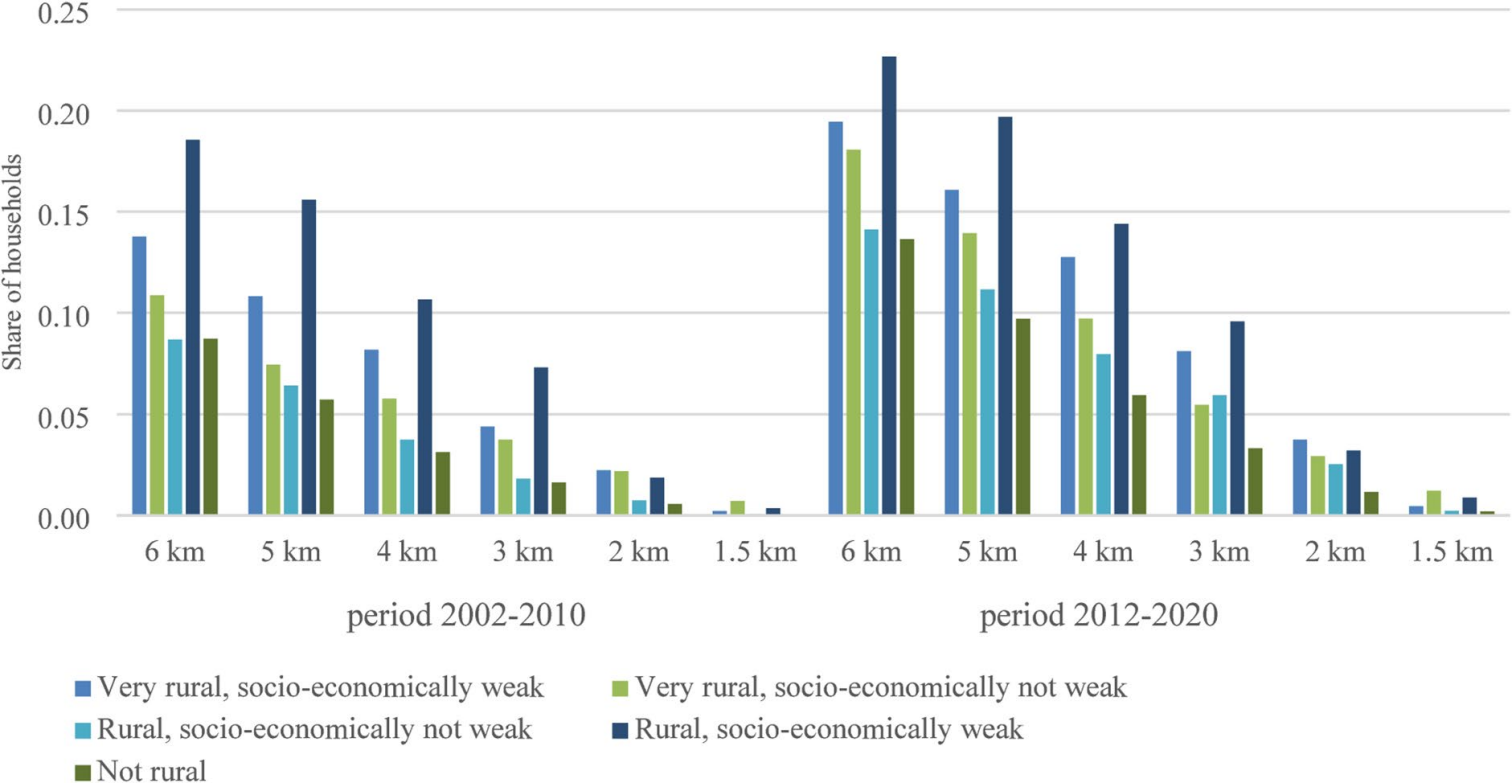
Share of households with wind turbines in their vicinity by type of rural area. Source: SOEP (v39) linked with Core Energy Market Data Register (MaSTR, v2023.04.01) with own corrections. Notes: Population estimates using the cross-sectional household population weights provided in SOEP. All onshore wind turbines are included.

Wind turbines within 6 km of residential homes have become increasingly common across all types of areas, including non-rural regions, as shown in Fig. 3. In socio-economically weak rural areas, 19% of households had at least one wind turbine within this distance in 2002–2010, increasing to 23% in 2012–2020. A comparable rise was observed in very rural socio-economically weak areas, where the share increased from 14% to 19%. In rural areas with stronger socio-economic profiles, the proportion rose from 11% to 19% over the same period. Wind turbine proximity is therefore not limited to very rural or socio-economically disadvantaged regions: in non-rural and more socio-economically advantaged rural areas, the share of households within 6 km of a turbine increased from 9% to 14%.

In the remainder of this section, we present results from a set of difference-in-differences fixed-effects panel estimations using matched samples of non-movers for our primary specification, focusing on turbines with hub height ≥ 50 m and treatment radii of 1.5 km to 6 km. The treatment group comprises individuals who experienced the construction of a wind turbine within a predefined radius of their residence, while the control group includes individuals without such exposure but with a wind turbine located between 10 km and 15 km from their home. The analysis compares two measurement points of health-related quality of life (HRQoL) to assess changes associated with turbine construction. Membership in the quasi-experimental treatment and control groups varies across specifications due to differences in the treatment definition and treatment radius. Descriptive statistics for the resulting analytical samples are reported in Supplementary Table S3.

Table 1 reports difference-in-differences estimates based on matched samples of residential non-movers. Treated individuals experience the commissioning of at least one qualifying turbine within the specified radius between $\:{t}_{0}$ and $\:{t}_{1}$, while control individuals have at least one turbine between 10 km and 15 km and no turbines within 10 km during the relevant observation window (see Methods). We do not find evidence of adverse average changes in mental HRQoL (MCS) across these radii. For physical HRQoL (PCS), most estimates are close to zero. The only coefficient reaching conventional statistical significance is the positive PCS estimate within 2 km (+ 3.577, *p* < 0.05), but this estimate does not retain significance after Benjamini-Hochberg FDR adjustment across the six PCS distance-band hypotheses (q > 0.05). Across the primary presence specifications in Table 1, no PCS or MCS coefficient remains statistically significant after FDR adjustment. We therefore interpret the isolated 2 km PCS estimate as suggestive rather than as robust evidence.

**Table 1:** Fixed effects difference-in-differences panel regressions of health-related quality of life (SF-12) on the presence of wind turbines with ≥ 50 m hub height within a radius of 1.5–6 km of residential homes (quasi-experimental sample).

	Mental health (MCS)				Physical health (PCS)				*N*
	β-coef.	SE	*p*-value	q-value	β-coef.	SE	*p*-value	q-value	
6 km	− 0.134	0.651	0.837	0.928	0.536	0.494	0.278	0.356	1688
5 km	− 0.495	0.789	0.531	0.928	0.252	0.603	0.676	0.666	1124
4 km	− 0.560	0.967	0.563	0.928	0.809	0.708	0.254	0.503	780
3 km	− 2.357	1.397	0.094	0.735	2.202	1.119	0.051	0.144	328
2 km	− 2.840	2.078	0.177	0.552	3.577*	1.772	0.048	0.144	128
1.5 km	− 0.622	2.368	0.794	0.928	0.253	2.238	0.911	0.911	76

Source: SOEP (v39) linked with Core Energy Market Data Register (MaSTR, v2023.04.01), with own corrections.

Notes: Cluster-robust standard errors (clustered at the individual level) are reported. Statistical significance is indicated at the 95% confidence level: * *p* < 0.05, ** *p* < 0.005, *** *p* < 0.001. q-values report Benjamini-Hochberg false discovery rate (FDR) adjusted p-values computed within this table separately for PCS and MCS across the reported distance bands. The BH procedure orders p-values within each family and sets $\:{q}_{i}={min}_{j\ge\:i\:}\left\{{m\:p}_{j}/j\right\}$, where $\:m$ is the number of hypotheses regarding that outcome (MCS or PCS) and $\:{p}_{j}$ denotes the $\:j$-th smallest p-value. Due to an insufficient number of matched treatment and control observations within a 1 km radius, the quasi-experimental analysis is restricted to observations located between 1.5 km and 6 km.

Wind turbines are frequently deployed in clusters rather than as isolated installations, and this pattern is also reflected in our quasi-experimental sample. Because cumulative exposure to multiple turbines may plausibly matter, we extend the analysis beyond binary presence indicators and examine whether turbine density within the treatment radius is associated with variation in HRQoL. Table 2 reports these results for our primary specification (turbines with hub height ≥ 50 m).

**Table 2:** Fixed effects difference-in-differences panel regressions of SF-12 on the number of wind turbines (quasi-experimental sample).

	Mental health (MCS)				Physical health (PCS)				*N*
	β-coef.	SE	*p*-value	q-value	β-coef.	SE	*p*-value	q-value	
6 km	− 0.177	0.209	0.396	0.510	0.090	0.193	0.641	0.690	1688
5 km	− 0.409	0.258	0.113	0.168	0.118	0.256	0.644	0.690	1124
4 km	− 0.697*	0.307	0.024	0.090	0.349	0.319	0.274	0.690	780
3 km	− 1.144**	0.349	0.001	0.006	0.340	0.474	0.474	0.690	328
2 km	− 3.015	1.873	0.112	0.168	3.094	1.659	0.067	0.402	128
1.5 km	− 0.622	2.368	0.794	0.794	0.253	2.238	0.911	0.911	76

Source: SOEP (v39) linked with Core Energy Market Data Register (MaSTR, v2023.04.01), with own corrections.

Notes: See Table 1.

We find evidence that higher turbine density is associated with lower mental HRQoL in some specifications. For turbines with hub height ≥ 50 m, each additional turbine within 3 km is associated with a decrease of -1.144 in MCS (*p* = 0.001, q = 0.006), and within 4 km with a decrease of -0.697 (*p* < 0.05, q = 0.090). The 3 km association remains statistically significant after FDR adjustment, whereas the 4 km association reaches conventional statistical significance but does not remain significant after FDR adjustment. We do not detect statistically significant associations between turbine density and PCS in these specifications.

Finally, we assess whether spatial proximity to wind turbines is associated with variation in HRQoL (Table 3). In the primary specification (hub height ≥ 50 m), point estimates are predominantly negative, but most are not statistically distinguishable from zero. Only one coefficient reaches conventional significance thresholds: physical HRQoL declines by -0.253 in the inverse-distance specification within 2 km (*p* < 0.05, q = 0.153). This estimate does not remain statistically significant after FDR adjustment. Across the inverse-distance specifications in Table 3, no PCS or MCS coefficient remains statistically significant after FDR adjustment. We therefore interpret the proximity-gradient evidence as limited in the main specification.

**Table 3:** Fixed effects difference-in-differences panel regressions of SF-12 on inverse distance to wind turbines (quasi-experimental sample).

	Mental health (MCS)				Physical health (PCS)				*N*
	β-coef.	SE	*p*-value	q-value	β-coef.	SE	*p*-value	q-value	
6 km	− 0.004	0.015	0.805	0.928	− 0.012	0.011	0.312	0.420	1688
5 km	0.002	0.022	0.945	0.928	− 0.003	0.018	0.880	0.873	1124
4 km	− 0.001	0.032	0.978	0.928	− 0.015	0.024	0.529	0.873	780
3 km	0.075	0.068	0.269	0.735	− 0.099	0.052	0.059	0.153	328
2 km	0.252	0.147	0.092	0.552	− 0.253*	0.115	0.032	0.153	128
1.5 km	0.017	0.189	0.928	0.928	0.030	0.188	0.873	0.873	76

Source: SOEP (v39) linked with Core Energy Market Data Register (MaSTR, v2023.04.01), with own corrections.

Notes: See Table 1.

As a robustness check, we re-estimate the primary matched non-mover DiD specifications, including additional time-varying individual-level covariates (age, education, employment status, marital status, personal and household income), district-level unemployment, and municipal road-traffic area share. Results are reported in Supplementary Table S4, with p-values and FDR-adjusted q-values shown alongside the coefficients. The covariate-adjusted models yield several conventionally significant estimates, particularly for MCS: turbine presence within 2 km, turbine density within 4 km, 3 km, and 2 km, and inverse distance within 2 km. For PCS, only the turbine-density coefficient within 3 km reaches conventional statistical significance. None of these estimates remains statistically significant after FDR adjustment. The substantive interpretation is therefore unchanged: evidence for adverse HRQoL changes is limited and should be interpreted cautiously.

Corresponding results on the three treatment definitions for alternative turbine definitions (all turbines, hub height ≥ 100 m, and installed capacity ≥ 500 kW) are reported in Supplementary Table S5, with p-values and FDR-adjusted q-values shown alongside the coefficients. The alternative definitions yield a small number of conventionally significant estimates. For all turbines and for turbines with capacity ≥ 500 kW, conventionally significant associations appear in the turbine-density specifications for MCS at 3 km and 4 km. For turbines with hub height ≥ 100 m, conventionally significant estimates appear for PCS in the presence specification at 6 km and in the inverse-distance specification at 6 km. However, none of these estimates remains statistically significant after FDR adjustment. These sensitivity analyses, therefore, do not alter the substantive interpretation of the primary results and are treated as exploratory robustness checks rather than as additional standalone findings.

We conducted two additional sensitivity analyses addressing spatial exclusion and pre-commissioning differences. First, we re-estimated the primary specifications using alternative ban radii of 9 km and 11 km, with corresponding control rings of 9–15 km and 11–15 km (Supplementary Table S6). These specifications do not produce FDR-robust evidence of adverse HRQoL changes. Second, we assessed pre-commissioning differences by comparing SF-12 changes between future treated and control groups before turbine commissioning (Supplementary Table S7). This diagnostic does not indicate a systematic pattern of pre-commissioning HRQoL differences: no coefficient remains statistically significant after FDR adjustment. One MCS estimate at the 1.5 km radius reaches conventional statistical significance, but this estimate is based on a very small treated sample and does not survive FDR adjustment.

## Discussion

Taken together, the results suggest that average changes in HRQoL among residential non-movers are generally small. The primary binary turbine-presence specifications do not provide FDR-robust evidence of adverse changes in either mental or physical HRQoL. The isolated positive PCS estimate within 2 km should not be interpreted as evidence of health benefits: it does not remain statistically significant after FDR adjustment and may reflect sampling variability, residual selection, differential attrition, or unobserved local changes coinciding with turbine commissioning.

The clearest pattern concerns cumulative exposure rather than turbine presence alone. In the primary turbine-density specification, higher turbine density within 3 km is associated with lower mental HRQoL and remains statistically significant after FDR adjustment. The corresponding 4 km estimate reaches conventional significance but is not FDR-robust. This pattern is consistent with the possibility that cumulative exposure may increase annoyance, stress, sleep disturbance, visual burden, or other perceptual pathways, even when the average effect of binary turbine presence is limited. Because we do not directly measure noise, shadow flicker, lighting, visibility, or annoyance, these mechanisms remain interpretive rather than directly tested.

Evidence for proximity-gradient effects is weaker. The inverse-distance specification shows one conventionally significant PCS estimate within 2 km, but this does not remain statistically significant after FDR adjustment, and the remaining proximity estimates are imprecise. Overall, the results therefore suggest that cumulative exposure may be more informative than proximity alone for understanding potential HRQoL differences around turbine commissioning, while also underscoring that the evidence is limited and specification-dependent.

The magnitudes should be interpreted in relation to the SF-12 scaling. PCS and MCS are norm-based scores with a standard deviation of 10 points; accordingly, a 1-point change corresponds to 0.1 standard deviations. The FDR-robust turbine-density estimate for MCS within 3 km corresponds to approximately − 0.11 standard deviations per additional turbine, while the conventionally significant 4 km density estimate corresponds to approximately − 0.07 standard deviations. The inverse-distance PCS estimate within 2 km corresponds to approximately − 0.03 standard deviations and is not FDR-robust. These effects are modest relative to overall variation in HRQoL, but density-related associations could accumulate in areas with multiple turbines.

## Conclusions

Using nationally representative longitudinal data from the SOEP linked to administrative wind turbine records, we estimate how first-time turbine commissioning is associated with changes in health-related quality of life among residential non-movers in Germany. Focusing on planning-relevant installations with hub height ≥ 50 m and proximity bands from 1.5 km to 6 km, the matched difference-in-differences results provide no FDR-robust evidence of adverse average HRQoL changes in the binary turbine-presence specifications. For physical HRQoL, the isolated positive PCS estimate within 2 km reaches conventional statistical significance but does not remain significant after FDR adjustment and should not be interpreted as evidence of health benefits. The clearest finding is instead in the turbine-density specification: higher turbine density within 3 km is associated with lower mental HRQoL and remains statistically significant after FDR adjustment. Other conventionally significant findings, including the turbine-density estimate within 4 km and the inverse-distance PCS estimate within 2 km, do not survive FDR adjustment and should therefore be interpreted cautiously.

Several limitations should be kept in mind when interpreting these findings. First, although the matched non-mover difference-in-differences design reduces confounding from stable individual characteristics and residential sorting, unobserved time-varying local factors may still bias estimates if they coincide with turbine commissioning (e.g., temporary construction-related activity, changes in local services or infrastructure, or other contemporaneous developments correlated with siting decisions^49)^. The covariate-adjusted models address part of this concern by accounting for observed time-varying individual and contextual characteristics, but they cannot rule out unobserved individual or local changes. The alternative ban-radius specifications show that the substantive interpretation is not driven by a single spatial exclusion rule, but changing the ban radius also changes the composition of the treated and control groups and therefore corresponds to a related, rather than identical, estimand. The pre-commissioning diagnostic assesses whether future treated and control groups already differed in SF-12 changes before commissioning, but it uses a different time window and cannot substitute for a full event-study analysis of anticipation, persistence, and longer-run adaptation. In particular, the biennial measurement of SF-12 and the limited number of treated observations at shorter radii restrict the scope for richer temporal diagnostics.

Second, restricting the quasi-experimental sample to residential non-movers strengthens internal validity by holding residential location constant around commissioning, but limits generalisability beyond residentially stable households. If turbine-sensitive individuals move in anticipation of commissioning or relocate shortly thereafter, estimated effects for non-movers may be attenuated. This concern should be interpreted in context: comparative OECD evidence characterises Germany as a relatively low-mobility setting, with residential mobility shaped by housing-market institutions, transaction costs, rental regulation and tenure status^50^. Prior SOEP-based research on wind turbine health impacts similarly focuses on non-movers, reports that only about 5% of individuals move in a given year, and treats mover-inclusive specifications as a robustness check rather than as the primary estimand^18^. Nevertheless, the present study does not model residential mobility as an outcome and therefore cannot assess whether turbine-sensitive individuals selectively leave exposed areas. Null or small average effects for residential non-movers, therefore, do not rule out effects for the full exposed population, but they characterise a substantial and policy-relevant group whose residential location remains stable around commissioning.

Third, exposure is defined using residence-centred distance buffers and registry-based turbine characteristics rather than direct measurements of noise, shadow flicker, lighting, visual intrusion, or annoyance. HRQoL is also a broad outcome and does not isolate specific pathways such as annoyance, stress, or sleep disturbance. Relatedly, the treatment definition captures first-time local exposure, defined as no turbines within 10 km at baseline and commissioning within the treatment radius by follow-up. The results should therefore not be generalised to repowering contexts or to longer-term exposure dynamics without further analysis.

Taken together, the findings suggest that cumulative exposure may matter more than proximity alone for certain dimensions of HRQoL, while average effects for residential non-movers are generally small. For siting and impact assessment, this implies that attention to minimum distance from the nearest turbine may be insufficient on its own; the number and spatial concentration of turbines around residential locations may also be relevant. Future research should combine longitudinal population data with objective environmental exposure measures, richer temporal designs, and explicit modelling of residential mobility to better characterise the conditions under which wind turbine deployment may affect population wellbeing.

## Supplementary Information

Below is the link to the electronic supplementary material.

Supplementary Material 1

## Data Availability

The analysis is based on data from the German Socio-Economic Panel (SOEP), which is available for scientific use from the German Institute for Economic Research (DIW Berlin) and is subject to data use agreements and data protection regulations. Access to SOEP data with geographic identifiers can be requested via the Research Data Centre at DIW Berlin. Administrative data on wind turbine locations and characteristics were obtained from the Core Energy Market Registry (MaStR) of the German Federal Network Agency. Archived versions of the MaStR are publicly available via the MaStR data download portal. Due to data protection requirements, the linked survey infrastructure dataset generated for this study cannot be made publicly available. Analyses combining individual survey data with geographic coordinates were conducted in a secure data environment at DIW Berlin. Researchers meeting the relevant access criteria may replicate the analyses by obtaining the underlying data from the respective providers and following the procedures described in the Methods. Analysis code is available from the authors upon reasonable request, subject to approval by the data providers and compliance with data protection regulations.

## Abbreviations

HRQoL: Health-related Quality of Life
SOEP: German SOcio-Economic Panel
MaStR: German Core Energy Market Register
